# Characterization of the *Paenibacillus beijingensis* DSM 24997 GtfD and its glucan polymer products representing a new glycoside hydrolase 70 subfamily of 4,6-α-glucanotransferase enzymes

**DOI:** 10.1371/journal.pone.0172622

**Published:** 2017-04-11

**Authors:** Joana Gangoiti, Lisa Lamothe, Sander Sebastiaan van Leeuwen, Christina Vafiadi, Lubbert Dijkhuizen

**Affiliations:** 1 Microbial Physiology, Groningen Biomolecular Sciences and Biotechnology Institute (GBB), University of Groningen, Groningen, The Netherlands; 2 Nestlé Research Center, Vers-Chez-Les-Blanc, Lausanne, Switzerland; University of Insubria, ITALY

## Abstract

Previously we have reported that the Gram-negative bacterium *Azotobacter chroococcum* NCIMB 8003 uses the 4,6-α-glucanotransferase GtfD to convert maltodextrins and starch into a reuteran-like polymer consisting of (α1→4) glucan chains connected by alternating (α1→4)/(α1→6) linkages and (α1→4,6) branching points. This enzyme constituted the single evidence for this reaction and product specificity in the GH70 family, mostly containing glucansucrases encoded by lactic acid bacteria (http://www.CAZy.org). In this work, 4 additional GtfD-like proteins were identified in taxonomically diverse plant-associated bacteria forming a new GH70 subfamily with intermediate characteristics between the evolutionary related GH13 and GH70 families. The GtfD enzyme encoded by *Paenibacillus beijingensis* DSM 24997 was characterized providing the first example of a reuteran-like polymer synthesizing 4,6-α-glucanotransferase in a Gram-positive bacterium. Whereas the *A*. *chroococcum* GtfD activity on amylose resulted in the synthesis of a high molecular polymer, in addition to maltose and other small oligosaccharides, two reuteran-like polymer distributions are produced by *P*. *beijingensis* GtfD: a high-molecular mass polymer and a low-molecular mass polymer with an average *M*_w_ of 27 MDa and 19 kDa, respectively. Compared to the *A*. *chroooccum* GtfD product, both *P*. *beijingensis* GtfD polymers contain longer linear (α1→4) sequences in their structure reflecting a preference for transfer of even longer glucan chains by this enzyme. Overall, this study provides new insights into the evolutionary history of GH70 enzymes, and enlarges the diversity of natural enzymes that can be applied for modification of the starch present in food into less and/or more slowly digestible carbohydrate structures.

## Introduction

The Glycoside Hydrolase family 70 (GH70) was originally defined for glucansucrase (GS) enzymes from lactic acid bacteria catalyzing the synthesis of α-glucans with various types of glucosidic linkages from sucrose [1,2]. According to the sequence-based CAZy classification system (http://www.cazy.org), GH70 family forms the GH-H clan, together with the GH13 and GH77 families of enzymes, both present in wide spectra of organisms and mainly catalyzing hydrolysis or/and transglycosylation of starch-like substrates [3,4]. Despite the diversity in substrate and reaction specificity among members of the GH-H clan, they all contain a catalytic (β/α)_8_-barrel, 4 catalytically important conserved sequence motifs, and a common α-retaining reaction mechanism [4–6]. These clear similarities in sequence, structure and reaction mechanism reflects their close evolutionary relatedness. Rather surprisingly, the three-dimensional structures of GSs revealed that these enzymes adopt a unique “U”-fold domain organization organized into five domains (A, B, C, IV and V) [7]. Except for domain C, all these domains are built-up from two discontinuous segments of the polypeptide chain. While domains IV and V are unique to GSs, domains A, B and C forming the catalytic core of GSs are also found in GH13 enzymes. However, in GSs domain A comprises a circularly permuted version of the catalytic (β/α)_8_-barrel found in GH13 and GH77 proteins [7–10]. As a consequence, the order of the conserved motifs (I-IV) of GH-H clan in GSs is II-III-IV-I, differing from the order I-II-III-IV characteristic of GH13 and GH77 enzymes. These structural differences led to the proposal that GSs evolved from an ancestor α-amylase by an evolutionary pathway based on the permutation per duplication model [7].

The evolutionary relationship between GH13 and GH70 families was further supported by the discovery of novel GH70 subfamilies of enzymes that appear to have an intermediate character between both families. First, the GtfB GH70 subfamily of enzymes was identified in several *Lactobacillus* strains [11,12]. The *L*. *reuteri* 121 GtfB is the main representative member of this subfamily of enzymes displaying a GS-like domain organization but unable to use sucrose as substrate. Instead, the *L*. *reuteri* 121 GtfB resembles GH13 α-amylase type of enzymes in using starch/maltodextrin substrates and acts as a 4,6-α-glucanotransferase (4,6-α-GTase), cleaving (α1→4) linkages and forming new consecutive (α1→6) linkages, resulting in the synthesis of linear isomalto/malto-polysaccharides (IMMP). These IMMP consist of (α1→6) glucan chains attached to the non-reducing ends of starch or malto-oligosaccharides fragments and are regarded as a new type of soluble dietary fiber [13]. Later, we have identified a second GH70 subfamily of enzymes (designated as GtfC) in *Exiguobacterium* and *Bacillus* strains and characterized the *Exiguobacterium sibiricum* 255–15 GtfC enzyme [14]. Biochemically, the *E*. *sibiricum* 255–15 GtfC is very similar to *L*. *reuteri* GtfB, both cleaving (α1→4) linkages and introducing (α1→6) linkages in linear chains. However, GtfC activity results in the synthesis of isomalto/malto-oligosaccharides (IMMO), instead of a modified polymer (IMMP). Surprisingly, GtfC enzymes lack the circular permutation of the (β/α)_8_ barrel characteristic of the GH70 family, and display an α-amylase like domain architecture, but with an extra continuous domain IV inserted in domain B. Despite of having a non-permuted domain organization, the clear sequence similarity shared between GtfC and GtfB 4,6-α-GTases led to the classification of GtfC protein sequences into the GH70 family.

The limited taxonomic distribution of GH70 family proteins recently was further expanded with the discovery of a novel GH70 enzyme (designated as GtfD) in the Gram-negative bacterium *Azotobacter chroococcum* NCIMB 8003 [15]. Regarding its domain organization, *A*. *chroococcum* GtfD is closely related to the GtfC type of enzymes possessing a non-permuted GH13-like architecture fold. The *A*. *chroococcum* GtfD enzyme also showed α1→6 transglucosylase activity on starch/maltodextrin substrates, but it displayed a unique product specificity. Instead of forming linear (α1→6) glucan chains, this enzyme was found to convert amylose into a branched and high molecular mass α-glucan with alternating (α1→4) and (α1→6) linkages. The structure of this polymer resembles that of the reuteran polymer produced by the *L*. *reuteri* 121 GS from sucrose [16,17], described as a dietary fiber with effect on satiety [18] and able to act as a bread improver [19] at the same time.

In this study we identified 4 new genes encoding putative GtfD-like 4,6-GTases in the genome sequences of taxonomically diverse plant-associated bacteria, forming a novel GH70 subfamily together with the previously characterized *A*. *chroococcum* GtfD enzyme. With the aim of further expanding the repertoire of starch-converting GH70 family enzymes, the GtfD enzyme of the plant-growth promoting rhizobacterium *Paenibacillus beijingensis* DSM 24997 was characterized. Our data shows that the *P*. *beijingensis* GtfD also is a 4,6-α-GTase producing a reuteran-like polymer, providing the first example of this novel reaction and product specificity in a Gram-positive bacterium. Clear differences between the products synthesized by the action of the *A*. *chroococcum* GtfD and *P*. *beijingensis* GtfD were found, enlarging the range of reuteran-like polymers that can be synthesized from amylose. Finally, the *A*. *chroococcum* GtfD and *P*. *beijingensis* GtfD isolated reuteran-like polymers, and the reaction mixtures obtained from starch incubations were subjected to *in vitro* digestibility studies with porcine pancreatin and rat intestinal powder extracts to evaluate the potential use of these enzymes for the production of slowly digestible and/or higher in fiber starchy foods.

## Materials and methods

### Bioinformatics

The *A*. *chroococcum* GtfD 4,6-α-glucanotransferase (Accession number: AJE22990.1) was used as the query sequence in BLASTp searches in NCBI (http://blast.ncbi.nlm.nih.gov/Blast) and IMG/ER (https://img.jgi.doe.gov/er/) public databases. The phylogenetic tree was constructed based on the alignment of representative GH70 and GH13 sequences identified by BLASTp using MEGA, version 6 [20]. A total of 72 complete protein sequences were aligned by MUSCLE using default parameters. The phylogenetic tree was obtained using the Maximum Likelihood method based on the JTT matrix model. Partial deletion of the positions containing alignment gaps and missing data was performed. Statistical confidence of the inferred phylogenetic relationships was conducted by performing 1,000 bootstrap replicates.

Prediction of a signal peptidase cleavage site was performed using Signal P4.1 server (http://www.cbs.dtu.dk/services/SignalP/). The theoretical *M*_w_ (molecular weight) of the *P*. *beijingensis* GtfD protein was predicted on ExPASy Compute pI/Mw (http://web.expasy.org/compute_pi/). Pairwise sequence comparisons of the *P*. *beijingensis* GtfD with the *L*. *reuteri* 121 GtfB, the *E*. *sibiricum* GtfC and the *A*. *chroococcum* GtfD protein sequences were performed using Jalview [21]. Multiple amino acid sequence alignments were generated with the Clustal Omega program (http://www.ebi.ac.uk/Tools/msa/clustalo/) and visualized by using Jalview [21].

### Cloning of the *P*. *beijingensis gtfD* gene

The 2241-bp DNA fragment coding for the full-length GtfD enzyme without its putative signal peptide-encoding sequence (amino acids 31 to 776) was amplified by PCR using Phusion DNA polymerase (Finnzyme, Helsinki, Finland) and the *P*. *beijingensis* chromosomal DNA (DSM 24997) as the template. The PCR primers used for amplifying the *gtfD* gene incorporated 5’ extensions (in bold) to facilitate the ligation-independent (LIC) cloning and were: PbF (5 ′ **CAGGGACCCGGT**GCGGAAAGCAATGCGAAAGG 3′) and PbR (5 ′ **CGAGGAGAAGCCCGGTTA**ATTGCTAAACCGTCTTAATGCTTTATTC 3′). The *gtfD* PCR product was cloned into a modified pET15b vector by ligation-independent cloning (LIC) as described before [14], resulting in a *gtfD* construct containing an N-terminal His6-tag cleavable by a 3C protease. The constructed expression vector pET15/PbGtfD was transformed into host *E*. *coli* BL21 Star (DE3). The construct was confirmed by sequencing (GATC, Cologne, Germany).

### Recombinant GtfD protein production in *E*. *coli* and purification

*Escherichia coli* BL21 Star (DE3) carrying pET15/PbGtfD was grown in 500-ml LB medium containing 100 μg ml^−1^ ampicillin in a rotary shaker (37°C, 220 rev min^−1^) to an optical density at 600 nm of 0.4–0.6. Expression of recombinant GtfD was induced by adding isopropyl-β-d-thiogalactopyranoside (IPTG) at a final concentration of 0.1 mM, and cultivation was continued at 16°C for 20 h. Cells were harvested by centrifugation (10,000 g x 20 min) and then disrupted with B-PER lysis reagent (Thermo Scientific, Pierce). After centrifugation (15,000 g x 20 min), the soluble GtfD protein was purified from the cell-free extract by His-tag affinity chromatography using Ni^2+-^nitrilotriacetate (Ni-NTA) as column material (Sigma-Aldrich). After washing the column with 25 mM Tris-HCl (pH 8.0), 1 mM CaCl_2_, bound proteins were eluted with 200 mM imidazole in the same buffer and the imidazole was removed by use of a stirred ultrafiltration unit (Amicon, Beverly, MA) with a 30,000 molecular weight cut off. Purity and homogeneity of the purified protein was analyzed by SDS-PAGE and the amount of protein in the enzyme solutions was routinely determined with a (Nanodrop 2000 spectrophotometer (Isogen Life Science, De Meern, The Netherlands).

### Enzyme activity assays

The initial activity of the purified *P*. *beijingensis* GtfD enzyme was determined by the iodine-staining assay using 0.125% (w v^-1^) amylose V (AVEBE, Foxhol, The Netherlands) as substrate [15,22]. This method monitors in time the decrease in absorbance at 660 nm of the α-glucan-iodine complex resulting from transglycosylation and/or hydrolytic activity. Enzymatic assays were carried out with 12 μg ml^-1^ of enzyme in 25 mM sodium phosphate buffer (pH 7.0) containing 1 mM CaCl_2_ at 50°C. One unit of activity is defined as the amount of enzyme converting 1 mg of substrate per min. The optimal pH and temperature were determined over the pH range of 4.5–10.0 and a temperature range of 35–60°C. Sodium citrate buffer (25 mM) was used for pH between 4.5 and 7.0, Sodium phosphate buffer (25 mM) for pH between 7.0 and 8.0, Tris-HCl buffer (25 mM) for pH between 8.0 and 9.0, and sodium bicarbonate buffer for pH between 8.0 and 9.0.

### Substrate specificity of *P*. *beijingensis* GtfD

The recombinant *P*. *beijingensis* GtfD enzyme (40 μg ml^-1^) was incubated separately with 25 mM sucrose (Acros), nigerose (Sigma-Aldrich), panose (Sigma-Aldrich), isomaltose (Sigma-Aldrich), isomaltotriose (Sigma-Aldrich), isomaltopentaose (Carbosynth), malto-oligosaccharides (MOS) with degrees of polymerization (DP) 2–7, and 0.6% (w v^-1^) amylose V (AVEBE, Foxhol, The Netherlands), and amylopectin (Sigma-Aldrich). Amylose V (AVEBE, Foxhol, The Netherlands) (1%, w v^-1^) was prepared as a stock solution in sodium hydroxide (1 M). Prior to use, the stock solution was neutralized by 7 M HCl and diluted to a concentration of 0.85% w v^-1^. All incubations were performed in 25 mM sodium phosphate buffer (pH 7.0) with 1 mM CaCl_2_ at 37°C for 24 h. Reactions were stopped by heating the samples to 100°C for 8 min. The progress of the reactions was assessed by thin-layer chromatography (TLC) and/or high-performance-anion-exchange chromatography (HPAEC).

### Thin layer chromatography and high performance anion exchange chromatography with pulsed amperometric detection analysis

Product mixtures from incubations with GtfD were spotted in 1-cm lines on silica gel 60 F254, 20 × 20 cm TLC sheets (Merck, Darmstadt, Germany). After drying, the TLC plates were developed in *n*-butanol:acetic acid:water (2:1:1, v/v) solvent system for 6 h. The bands were visualized with orcinol/sulfuric acid staining and compared with a simultaneous run of a mixture of glucose and MOS (DP2 to DP7).

Carbohydrate samples were diluted 3:100 in DMSO and analyzed by HPAEC on an ICS3000 workstation (Dionex, Amsterdam, The Netherlands), equipped with a CarboPac PA-1 column (Thermo Scientific, Amsterdam, The Netherlands; 250 x 2 mm) and an ICS3000 pulsed amperometric detection (PAD) system. The injection volume of each sample was 5 μl, and the oligosaccharides were separated by using a linear gradient of 10–240 mM sodium acetate in 100 mM NaOH over 57 min at a 0.25 ml min^-1^ flow rate. The identity of the peaks was assigned using commercial oligosaccharide standards.

### Production and structural analysis of the products from amylose incubation with GtfD

Amylose V (0.6% w v^-1^) was incubated with purified GtfD (0.2 mg) under the conditions described in “Substrate specificity of *P*. *beijingensis* GtfD”. After incubation for 24 h at 37°C, the reaction was stopped by transfer to 100°C for 8 min. The HMM and LMM polysaccharide fractions were isolated by size-exclusion chromatography on a Superdex S-200 (10 x 300 mm; GE-Healthcare) using 25 mM ammonium bicarbonate as eluent at a flow rate of 0.5 ml min^-1^. For comparison the amylose-derived *A*. *chroococcum* GtfD polymer was also produced and isolated as described before [15].

### NMR spectroscopy

Resolution-enhanced 1D/2D ^1^H and ^13^C NMR spectra were recorded in D_2_O on a Varian Inova-500 spectrometer (NMR center, University of Groningen, The Netherlands) at probe temperature of 298 K. Prior to analysis, samples were exchanged twice in D_2_O (99.9 at% D, Cambridge Isotope Laboratories, Inc., Andover, MA) with intermediate lyophilization, and then dissolved in 0.6 ml D_2_O. One-dimensional 500-*MHz* ^1^H NMR spectra were recorded at a 4 000 *Hz* spectral width and 16k complex points, using a WET1D pulse to suppress the HOD signal. Two-dimensional ^1^H-^1^H spectra (COSY, TOCSY MLEV17 30, 50, and 150 ms, and ROESY 300 ms) were recorded with 4 000 *Hz* spectral width, collecting 200 increments. In case of TOCSY spectra 2 000 complex data points were collected, for COSY and ROESY spectra 4 000 complex data points were used. 2D ^13^C-^1^H NMR spectra were recorded in 128 increments of 2 000 complex points with 4000 *Hz* spectral width in *t2* and 10 000 *Hz* in *t1*. All NMR data were processed using MestReNova 5.3 (Mestrelabs Research SL, Santiago de Compostella, Spain). Manual phase correction and Whittacker smoother baseline correction were applied to all spectra. Chemical shifts (*δ*) are expressed in ppm with reference to internal acetone (*δ* 2.225 for ^1^H and *δ* 31.08 for ^13^C).

### HPSEC analysis

Molecular mass distribution characterization of the products mixtures was performed as described before [15,22]. Briefly, samples were dissolved at a concentration of 4 mg ml^-1^ in DMSO-LiBr (0.05 M), filtered through a 0.45 μm PTFE membrane and analyzed by HPSEC coupled on-line with a multi angle laser light scattering detector (SLD 7000 PSS, Mainz), a viscometer (ETA-2010 PSS, Mainz) and a differential refractive index detector (G1362A 1260 RID Agilent Technologies). Separation was carried out by using three PFG-SEC columns with porosities of 100, 300 and 4000 Å, coupled with a PFG guard column. DMSO-LiBr (0.05 M) was used as eluent at a flow rate of 0.5 ml min^-1^. The system was calibrated and validated using a standard pullulan kit (PSS, Mainz, Germany) with *M*_w_ ranging from 342 to 805 000 Da. The specific RI increment value (dn/dc) was also measured by PSS and was 0.072 ml g^−1^ (private communication with PSS). The multiangle laser light scattering signal was used to determine the molecular masses of the amylose and the HMM polysaccharides generated by the *A*. *chroococcum* and *P*. *beijingensis* GtfD enzymes. The dn/dc value for these polysaccharides in this system was taken to be the same as for pullulan. The molecular mass of the *P*. *beijingensis* LMM polymer was determined by universal calibration method. WinGPC Unity software (PSS, Mainz) was used for data processing. Measurements were performed in duplicate.

### Methylation analysis

Methylation analysis was performed as described earlier [23]. Briefly, the isolated polysaccharides (∼5 mg) were per-methylated using CH_3_I and solid NaOH in DMSO, and subsequently hydrolyzed with trifluoroacetic acid. The partially methylated monosaccharides generated were reduced with NaBD_4_. The resulting partially methylated alditols were per-acetylated using pyridine:acetic anhydride (1:1 v/v) at 120°C yielding mixtures of partially-methylated alditol acetates, which were analyzed by GLC-EI-MS and GLC-FID as described [23].

### Product analysis with hydrolytic enzymes

*P*. *beijingensis* GtfD isolated HMM and LMM polysaccharides, reuteran GtfA polymer, IMMP GtfB polymer, and *A*. *chroococcum* GtfD polymer (5 mg ml^-1^) were subjected to enzymatic degradation using excess α-amylase (*Aspergillus oryzae* α-amylase; Megazyme), dextranase (*Chaetomium erraticum*; Sigma-Aldrich), or pullulanase M1 (*Klebsiella planticola*; Megazyme). Reactions were performed in 50 mM sodium acetate buffer pH 5.0 for 48 h at 37°C. In all cases, the degree of degradation was assessed by TLC and/or HPAEC. In parallel, enzymatic hydrolysis of amylose, dextran and pullulan, were carried out and used as positive controls for the α-amylase, dextranase and pullulan digestions, respectively. Under the reaction conditions used, these polysaccharides were completely hydrolyzed.

### *In vitro* digestibility of the products synthesized by the *P*. *beijingensis* and *A*. *chroococcum* GtfD enzymes from amylose and wheat starch

The isolated amylose–derived polymers and wheat starch-derived products resulting from the activity of the *P*. *beijingensis* GtfD and the *A*. *chroococcum* GtfD enzymes were subjected to *in vitro* simulations of human small intestinal digestion. For the preparation of the GtfD enzyme-modified wheat starch products, wheat starch was gelatinized (Sigma-Aldrich) by heat treatment (90°C, 10 min) at a concentration of 0.6% w v^-1^, and subsequently incubated separately with *P*. *beijingensis* GtfD enzyme and *A*. *chroococcum* GtfD enzyme (4.6 μg ml^-1^) in MilliQ water containing 1 mM CaCl_2_ at 37°C for 24 h. Reactions were stopped by heating the samples to 95°C for 6 min, and subsequently lyophilized. The amylose–derived polymers were prepared and isolated as described in “Production and structural analysis of the products from amylose incubation with GtfD”.

For the preparation of the digestive enzymes, pancreatin from porcine pancreas (Sigma-Aldrich) and intestinal acetone powders from rat (Sigma-Aldrich) were suspended separately at a concentration of 40 mg ml^-1^ in 10 mM PBS buffer solution (pH 6.8), vortexed and sonicated for 7 min on ice. After centrifugation (10,000 x g, 30 min, 4°C), the supernatants were collected, and the protein content as well as enzyme activities were measured. Protein concentration was determined by the BCA (Bicinchoninic Acid) kit (Sigma-Aldrich) using bovine serum albumin as standard [24]. The activity of the extracted enzymes was determined using 0.5% (w v^-1^) potato starch (Sigma-Aldrich) as substrate by measuring the amount of glucose released. Enzymatic assays were performed with 100 U ml^− 1^ of the extracted proteins in 10 mM PBS buffer (pH 6.8) at 37°C with constant stirring. After 10 min, the reactions were stopped by incubation at 100°C for 10 min, and the glucose released was determined at 505 nm using the Autokit Glucose assay (Wako Diagnostics). One unit of enzyme activity was defined as the amount of protein required to hydrolyze 1 μg of glucose from soluble potato starch.

The different α-glucan samples (1 mg ml^-1^) were incubated with a combination of 100 U ml^-1^ of each of the extracted digestive enzyme solutions in 10 mM PBS buffer pH 6.8 at 37°C with constant stirring in a total volume of 1.37 ml. Samples of 500 μl were taken after 20, 60, and 120 min, and subsequently transferred into a tube containing 1.5 mL of 90% w v^-1^ aqueous ethanol. These samples were stored at 4°C, centrifuged (10,000 x g, 10 min), and the supernatants used to quantify the amount of free glucose resulting from the hydrolytic activity of the digestive enzymes on the α-glucans by a glucose quantification kit (Autokit Glucose, Wako Diagnostics). Controls without the digestive enzymes were carried out in parallel to correct the impact of enzyme solution on the absorbance. The amount of hydrolyzed α-glucan was expressed as the percentage of the initial α-glucan product that was hydrolyzed into glucose. Measurements were performed in duplicate.

## Results and discussion

### Identification of GtfD-like 4,6-α-glucanotransferases in taxonomically diverse plant-associated bacteria

**Fig 1:**
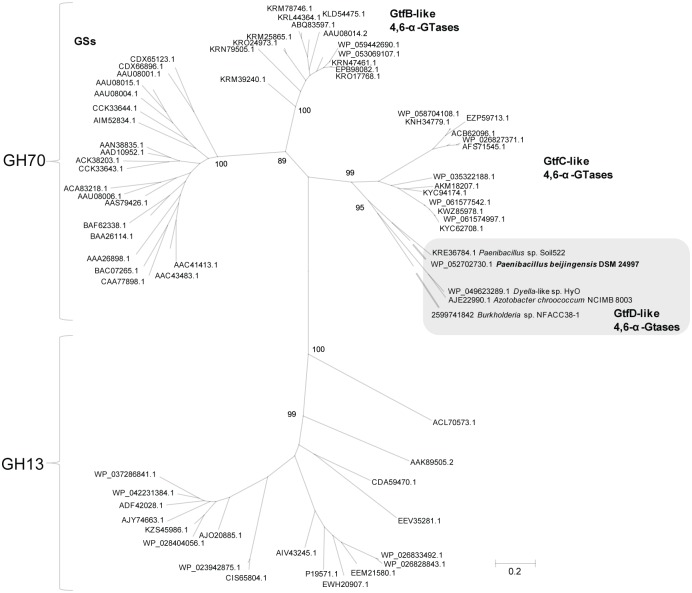
Unrooted phylogenetic tree of representative family GH13 and GH70 protein sequences identified by BLASTp searches using the *A*. *chroococcum* GtfD 4,6-α-GTase protein as query.

Proteins homologous to the *A*. *chroococcum* GtfD were identified in the genomes of numerous taxonomically diverse bacteria by BLASTp searches within the NCBI and IMG-ER platforms (S1 Table). These searches revealed that the *A*. *chroococcum* GtfD shows 71, 48, 46 and 45% amino acid sequence identity to the hypothetical GH70 enzymes encoded by the *Dyella*-like sp. HyOG (Genbank accession WP_049623289.1), *Paenibacillus beijingensis* DSM 24997 (Genbank accession WP_052702730.1), *Burkholderia* sp. NFACC38-1 (IMG/ER Gene ID 2599741842), and *Paenibacillus* sp. Soil522 (Genbank WP_056638435.1), respectively. Besides, 23 homologs of this enzyme present in *Bacillus* and *Exiguobacterium* strains were identified, sharing more than 40% and 39% of identity, respectively. From these proteins, only the GtfC 4,6-α-GTase enzyme encoded by the psychrotrophic bacterium *E*. *sibiricum* 255–15 has been biochemically characterized [14]. Also, in the recently elucidated genome of the thermophile *Geobacillus* sp. 12AMOR1 we identified a protein 42% identical to the *A*. *chroococcum* GtfD enzyme. The next hits obtained were (putative) GtfB-like 4,6-α-GTases, followed by (putative) glucansucrases, all of them encoded by lactic acid bacteria. The sequences of (putative) GH13 α-amylases present in diverse bacteria were retrieved as the last hits of the BLASTp searches. Phylogenetic analysis of representative GH70 and GH13 proteins identified by BLASTp is presented in Fig 1. The *A*. *chroococcum* GtfD and its homologues encoded by *Dyella*-like sp. HyOG, *P*. *beijingensis* DSM 24997, *Burkholderia* sp. NFACC38-1, and *Paenibacillus* sp. Soil522, form a clearly distinct group. This novel cluster of proteins designated as GtfD, is closely related to the GtfC subfamily of enzymes present in *Bacillus*, *Geobacillus* and *Exiguobacterium* strains. GtfC and GtfD type of enzymes are positioned between GtfB-like GH70 proteins and GH13 α-amylases. This location likely reflects the evolutionary intermediate position of these enzymes between GH70 and GH13 families. As reported before, *E*. *sibiricum* GtfC and *A*. *chroococcum* GtfD display clear sequence similarity with GtfB-like 4,6-α-GTases, but present a GH13 α-amylase-like domain organization [14,15].

The evolutionary history was inferred by using the Maximum Likelihood method based on the JTT matrix-based model. The scale bar corresponds to a genetic distance of 0.2 substitutions per position. The bootstrap values adjacent to the main nodes represent the probabilities based on 1000 replicates. The protein sequences are annotated by their GenBank Accession number, except for the *Burkholderia* sp. NFACC38-1 GtfD-like 4,6-α-GTase protein sequence that was only identified in the IMG/ER database and is labeled with its IMG/ER Gene ID. The names of the bacterial species are provided in S1 Fig. The GtfD subgroup of enzymes is highlighted with a grey background. The *Paenibacillus beijingensis* DSM 24997 GtfD-like 4,6-α-GTase is shown in bold.

The growing number of genome sequencing initiatives has resulted in the identification of this small GtfD cluster of GH70 enzymes and is rapidly expanding the number of GSs, GtfB and GtfC type of enzymes available in public databases. Except for the GtfD protein of the Gram-negative *A*. *chroococcum* strain NCIMB 8003, up to date the distribution of GH70 enzymes appeared to be restricted to Gram-positive bacteria grouped in the Class *Bacilli* of the low GC phyla of Firmicutes. GSs and GtfB-like 4,6-α-GTases are exclusively found in lactic acid bacteria, whereas GtfC-like 4,6-α-GTases, are limited to three bacterial genera belonging to the order *Bacillales* such as *Exiguobacterium*, *Bacillus* and *Geobacillus*. With a few exceptions, the phylogeny of these GS, GtfB and GtfC GH70 enzymes is generally in agreement with their bacterial origin [6]. Surprisingly, the GtfD subgroup of enzymes has originated from various distinct taxonomic groups of Gram-negative and Gram-positive bacteria suggesting that the *gtfD* encoding genes were acquired through horizontal transfer. Three of the identified GtfD-like proteins are encoded by diverse Gram-negative Proteobacteria: *Azotobacter chroococcum* and *Dyella*-like sp. HyOG belong to the *Pseudomonadales* and *Xanthomonadales* orders in the γ-proteobacteria, respectively; *Burkholderia* sp. NFACC38-1 is classified into the β-proteobacteria class. Besides, 2 GtfD proteins were also identified in Gram-positive *Paenibacillus* species regarded as taxonomically close to the *Bacillus* genus. Some of these bacteria are part of soil and rhizosphere communities, while others colonize internal plant tissues (S1 Table). *A*. *chroococcum* NCIMB 8003 is a well-known heterotrophic soil-dwelling bacterium of ecological importance due to its ability to promote plant growth by providing fixed nitrogen to the plant [25]. *P*. *beijingensis* DSM 24997 and *Paenibacillus* sp. Soil522 were isolated from rhizosphere soils of jujube and *Arabidopsis thaliana*, respectively [26,26,27]. *Burkholderia* sp. NFACC38-1, instead, is a root-associated endophyte of switchgrass. *Dyella*-like sp. HyOG was isolated from the gut of the grapevine yellows disease insect vector *Hyalesthes obsoletus*. This bacterium settles inside the phytoplasma's infected grapevine and reduces the grapevine yellows disease symptoms (NCBI Bioproject accession no: PRJNA286074). The presence of GtfD enzymes in diverse members of plant-associated ecosystems suggests that the products of these enzymes confer adaptability to these environments. In plant-associated bacteria, the production of exopolysaccharides (EPS) has been shown to be essential for bacterial attachment to plant surfaces or to other bacteria, and for biofilm formation, thereby promoting plant colonization [28]. GtfD type of enzymes may have similar physiological roles in the development of plant-microbe interactions.

In view of the unique and unexplored origin of the GtfD subgroup of GH70 enzymes, the GtfD enzyme from *P*. *beijingensis* DSM 24997 was selected for further study as the first example of a GtfD-like protein encoded by a Gram-positive bacterium. A functional annotation of the *P*. *beijingensis* DSM 24997 genome revealed that this bacterium presents the typical phenotypic features commonly found in plant growth-promoting rhizobacteria (PGPR) such as the ability to fix atmospheric N_2_, and as a consequence has a great potential for agricultural applications [27].

### Primary sequence analysis of the *P*. *beijingensis* GtfD enzyme

**Fig 2:**
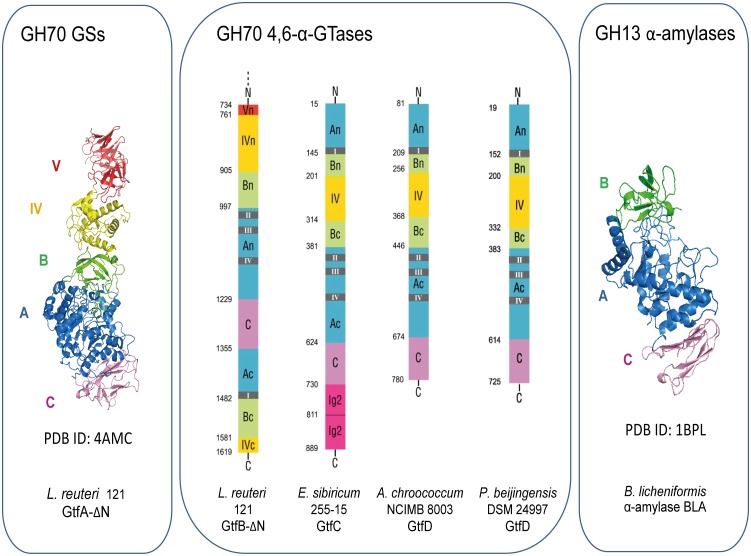
Predicted domain arrangement of representative members of GH70 and GH13 families with focus on GH70 4,6-α-GTase enzymes. The crystal structures of the *L*. *reuteri* 121 GtfA glucansucrase (left) [10] and the *B*. *licheniformis* α-amylase (right) [29] are included. Domains A, B, C, IV and V are highlighted in blue, green, magenta, yellow and red, respectively. Ig2-like domains are colored in pink. The amino acid residue numbers represent the start of each domain. Conserved regions I-IV are indicated by grey rectangles. Domains A, B, C and IV were assigned in *P*. *beijingensis* GtfD by sequence comparison with *L*. *reuteri* 121 GtfB.

The identified *P*. *beijingensis* GtfD protein sequence consists of 776 amino acids and contains a putative secretion signal peptidase cleavage site between amino acids 30 and 31, in accordance with the extracellular location of GH70 enzymes. The *P*. *beijingensis* GtfD exhibited the highest sequence identity with the GtfD protein identified in *Paenibacillus* sp. Soil522 (63% identity) and shared 48–52% of identity with the GtfD enzymes encoded by Gram-negative bacteria. The domain organization of the *P*. *beijingensis* GtfD resembles that of *E*. *sibiricum* GtfC and *A*. *chroococcum* GtfD enzymes, regarded as structurally evolutionary intermediates between GH13 and GH70 families (Fig 2). Consequently, this enzyme displays a GH13-like domain arrangement with a non-permuted catalytic (β/α)_8_ barrel, but possesses an extra domain IV inserted in domain B. Similar to *E*. *sibiricum* GtfC and *A*. *chroococcum* GtfD, this enzyme lacks the variable N-terminal domain and the domain V typically found in GH70 GSs and GtfB homologues. Also, the Ig2-like domains identified in the C-terminal part of some GtfC-like proteins, appeared to be absent from *P*. *beijingensis* GtfD, as observed in case of the *A*. *chroococcum* GtfD.

On the basis of sequence alignments the four conserved regions of clan GH-H were identified in *P*. *beijingensis* GtfD and compared with those in other GH70 proteins. In accordance with its non-permuted domain organization, the order of these four conserved regions in *P*. *beijingensis* GtfD and other GtfD-like proteins is I-II-III-IV, instead of the permuted order II-III-IV-I characteristic of GH70 glucansucrases and GtfB-like 4,6-α-GTases. The seven amino acid residues that are fully conserved in motifs I to IV of all GH70 family members are also found in all GtfD-like proteins (Table 1). Among these seven residues, the nucleophile, the general acid/base and the transition state stabilizer of the catalytic triad were identified as Asp409, Glu442 and Asp512 in *P*. *beijingensis* GtfD (*P*. *beijingensis* GtfD numbering is used throughout unless indicated otherwise), respectively. These residues are also conserved in most GH13 family members with the exception of Gln151, which is replaced by an His residue in GH13 proteins (His140, BSTA *Bacillus stearothermophilus* α-amylase numbering) [30]. Compared to other GH70 family proteins, the conserved regions I to IV of the GtfD-like proteins showed the highest similarity with those of the GtfC subfamily of enzymes. Otherwise, a number of functionally significant residues, in particular residues forming the donor/acceptor subsites in GSs, are conserved across GtfB, GtfC and GtfD homologues with (putative) 4,6-α-GTase activity. Of note is the presence of a Tyr in GtfB, GtfC and GtfD proteins replacing the subsite +1/+2 Trp residue conserved in almost all GSs (W1065 in GTF180-ΔN). A conserved Tyr is also present in the *Lactobacillus fermentum* GtfB active on (α1→4 glucans), but displaying (α1→3) linkage specificity [31] suggesting that this residue may be considered a “sequence fingerprint” of GH70 proteins active on starch and maltodextrins. Also, GtfB, GtfC and GtfD type of enzymes display high conservation in the amino acids following the transition state stabilizer located in motif IV. Specifically, all these proteins have one amino acid gap between the second and third residue downstream the transition state stabilizer and possess a conserved Gln at position 513, known to be important for the correct binding of the sugar moiety at subsite +2 in glucansucrases. However, GtfD proteins differ from GtfB and GtfC homologues by the presence of an His at position 515, replacing the Lys found in most of the GtfB- and GtfC- like proteins, whereas GSs present an invariantly conserved Gln at this position. This unique sequence feature could thus be regarded as a sequence fingerprint for the reaction and product specificity displayed by GtfD type of enzymes (see below). In region II, the subsite +1 Asn residue conserved in most GSs and (putative) GtfB-like 4,6-α-GTs is replaced by an His in GtfC and GtfD proteins (His413 in *P*. *beijingensis* GtfD). Interestingly, only the *L*. *fermentum* GtfB acting as a 4,3-α-glucanotransferase shows unique variations in residues 413, 513 and 516 contributing to the substrate binding subsites, suggesting that these residues are important for the linkage specificity.

**Table 1:** Alignment of conserved motifs I-IV of GH70 family enzymes. (A) (putative) GtfD-like 4,6-α-GTase enzymes, (B) (putative) GtfC-like 4,6-α-GTase enzymes, (C) (putative) GtfB-like enzymes, and (D) sucrose-active enzymes. The seven strictly conserved amino acid residues in GH70 enzymes (numbered 1 to 7 above the sequences) are also conserved in GtfD-like proteins. Amino acids that constitute the catalytic triad are highlighted in bold and lightly shaded. Residues forming acceptor subsites -1, +1 and +2 in Gtf180-ΔN [7] are indicated in green, red and blue, respectively. Abbreviations at the bottom: NU = nucleophile, A/B = general acid/base, TS = transition state stabilizer. ^a^ The protein sequences are annotated by their GenBank Accession number, except for the *Burkholderia* sp. NFACC38-1 GtfD-like protein sequence that is labeled with its IMG/ER Gene.

Bacterial strain	Accession numbers^a^	Specificity		Motif I		Motif II		Motif III		Motif IV
**A**				**1 2**		**3 4**		**5**		**67**
*Paenibacillus beijingensis*	WP_045672861.1	4,6-α-GTase	145	VDLVPNQ	405	GFRIDAASHYN	437	HLSYIESYTDN	507	FVMNHDQE-HNGIKG
*Azotobacter chroococcum* NCIMB 8003	AJE22990.1	4,6-α-GTase	202	VDVVPNQ	467	GFRIDAASHIN	500	HLSYIESYVTQ	567	FVNNHDQE-HNILVT
*Bacterium of Hyalesthes Obsoletus*	WP_049623289.1	ND	212	VDLVPNQ	477	GFRIDAASHIN	510	HLSYIESYVTA	577	FVNNHDQE-HNLLAG
*Burkholderia sp*. NFACC38-1	2599741842	ND	103	ADIVPNQ	362	GFRFDAAGHYN	394	HLSVIESYVDP	465	FVTNHDQE-HNVIAK
*Paenibacillus sp*. *Soil522*	WP_056638435.1	ND	149	EDLVPNQ	403	GFRIDAASHLN	435	HLSFIESYTDN	505	FVNNHDQE-HNAIKP
**B**										
*Exiguobacterium sibiricum* 255–15	ACB62096.1	4,6-α-GTase	138	MDLVPNQ	403	GFRIDAASHYD	433	HLSYIESYKSE	504	FVNNHDQE-KNRVNQ
*Exiguobacterium undae*	WP_028105602.1	ND	138	MDLVPNQ	403	GFRIDAASHYD	433	HLSYIESYKSE	504	FVNNHDQE-KNRVNQ
*Exiguobacterium antarcticum* B7	AFS71545.1	ND	138	MDLVPNQ	403	GFRIDAASHYD	433	HLSYIESYKSE	504	FVNNHDQE-KNRVNQ
*Exiguobacterium acetylicum*	WP_029342707.1	ND	138	MDLVPNQ	403	GFRIDAASHYD	433	YLSYIESYKTE	503	FVNNHDQE-KNRVNQ
*Bacillus kribbensis*	WP_035322188.1	ND	130	EDLVPNQ	397	GFRIDAASHYD	429	HLSYIESYSNV	491	FVNNHDQE-KNRVNN
*Bacillus coagulans* DSM1	AJH79253.1	ND	128	EDLVPNQ	394	GFRIDAAGHYD	426	HLSYIESYQSA	497	FVTNHDQE-KNRINN
*Bacillus sporothermodurans*	KYC94174.1	ND	140	EDLVPNQ	408	GFRVDAASHYD	440	HLSYIESYSSA	511	FVTNHDQE-KNRINN
*Geobacillus sp*. *12AMOR1*	AKM18207.1	ND	140	LDLVPNQ	409	GFRIDAATHFD	441	HLSYIESYTSK	512	FVNNHDQE-KNRVNT
**C**										
*Lactobacillus reuteri* 121 (GtfB)	AAU08014.2	4,6-α-GTase	1478	EDIVMNQ	1011	GFRVDAADNID	1048	HLSYNEGYHSG	1120	FVTNHDQR-KNLINR
*Lactobacillus reuteri* ML1 (ML4)	AAU08003.2	4,6-α-GTase	1479	EDIVMNQ	1012	GFRVDAADNID	1049	HLSYNEGYHSG	1121	FVTNHDQR-KNLINR
*Lactobacillus reuteri* DSM 20016 (GtfW)	ABQ83597.1	4,6-α-GTase	1215	EDLVMNQ	748	GFRVDAADNID	785	HLVYNEGYHSG	858	FVTNHDQR-KNVINQ
*Pediococcus pentosaceus* IE-3	CCG90643.1	ND	841	EDIVMNQ	380	GFRIDAADNID	417	HLSYNEGYHSG	489	FVTNHDQR-KNLINS
*Lactobacillus acidipiscis* KCTC 13900	WP_035631372.1	ND	765	VDMVMNQ	296	GFRNDAADNID	333	HLVYNEGYHSG	406	FVTNHDQR-KNVINQ
*Lactobacillus panis* DSM 6035	KRM25865.1	ND	1455	EDLVMNQ	988	GFRVDAADNVD	1025	HLVYNEGYHSD	1097	FVTNHDQR-KNLINQ
*Leuconostoc mesenteroides*	WP_059442690.1	ND	711	EDIVMNQ	252	GFRIDAADHID	289	HLIYNEGYRSG	360	FVTNHDQR-ANLING
*L*. *fermentum* NCC 2970	AOR73699	4,3-α-GTase	1446	EDIVMNQ	983	GFRIDAADDMD	1020	HLSYNEGYGPG	1092	YVTNHDIR-NNLING
**D**										
*Lactobacillus reuteri* 180 (Gtf180)	AAU08001.1	Dextransucrase	1503	ADWVPDQ	1021	GIRVDAVDNVD	1058	HINILEDWGWD	1131	FVRAHDSNAQDQIRQ
*Lactobacillus reuteri* 121 (GtfA)	AAU08015.1	Reuteransucrase	1508	ADWVPDQ	1020	SVRVDAPDNID	1056	HINILEDWNHA	1128	FVRAHDNNSQDQIQN
*Streptococcus mutans* SI (GtfSI)	BAA26114.1	Mutansucrase	954	ADWVPDQ	473	SIRVDAVDNVD	510	HLSILEAWSYN	583	FIRAHDSEVQDLIRD
*Leuconostoc mesenteroides* NRRL-1355	CAB65910.2	Alternansucrase	1168	ADWVPDQ	631	GIRVDAVDNVD	668	HLSILEDWNGK	762	FVRAHDYDAQDPIRK
*Leuconostoc citreum* NRRL B-1299	CDX66820.1	(1→2) Branching sucrase	2688	ADVVDNQ	2206	SIRIDAVDFIH	2243	HISLVEAGLDA	2317	IIHAHDKGVQEKVGA
*Leuconostoc citreum* NRRL B-742	CDX65123.1	(1→3) Branching sucrase	1182	ADFVANQ	667	SMRIDAISFVD	704	HISIVEAPKGE	783	IVHAHDKDIQDTVIH
						**NU**		**A/B**		**TS**

### Purification and biochemical properties of the *P*. *beijingensis* GtfD enzyme

Recombinant *P*. *beijingensis* GtfD without its peptide signal sequence (amino acids 31–776) was expressed in soluble form at high levels and purified to homogeneity from *E*. *coli* BL21 star (DE3) by His-tag affinity chromatography yielding 50 mg of pure protein per liter of culture. SDS-PAGE analysis of the pure enzyme revealed the appearance of a single ~ 80-kDa protein band (Data not shown), consistent with the predicted molecular mass deduced from its amino acid sequence (85 kDa). The effects of pH and temperature on enzyme activity were determined by the amylose-iodine assay. The GtfD enzyme of *P*. *beijingensis* displayed its maximum activity at pH 7.0 and 50°C. A higher optimum temperature value was reported for the *A*. *chroococcum* GtfD enzyme (65°C), whereas no significant differences in the optimal pH value existed between both GtfD enzymes [15]. In contrast, the GtfB 4,6-α-GTases characterized from *Lactobacillus* strains have been reported to show significantly more acidic optimum pH values of 4.5 and 5 reflecting their adaptation to the gastrointestinal tract [12,22]. The specific total activity value of the *P*. *beijingensis* GtfD in 25 mM sodium phosphate buffer, pH 7.0, containing 1 mM CaCl_2_, and at 50°C was 6.3 ± 0.17 U mg^-1^, and is similar to that of *A*. *chroococcum* GtfD (at pH 6.5 and 50°C), namely 6.6 ± 0.05 U mg^-1^. Thus, both GtfD enzymes exhibited remarkably higher total specific values than those determined for the *L*. *reuteri* GtfB and the *E*. *sibiricum* GtfC 4,6-α-GTase, whose specific activity values were 2.8 U mg^-1^ and 2.2 U mg^-1^, respectively (at 40°C and pH 5 and 6, respectively) [14].

### Substrate and product specificity

**Fig 3:**
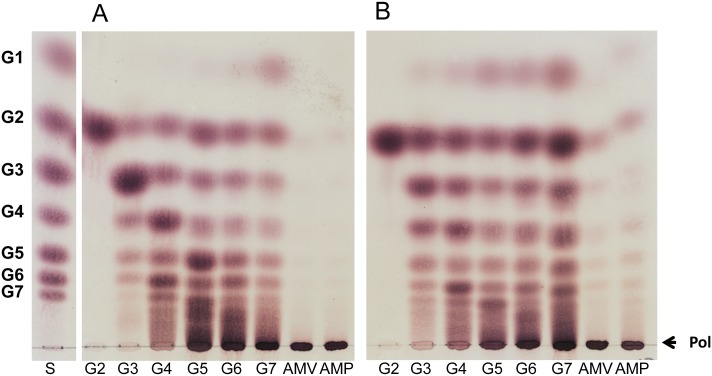
TLC analysis of the products synthesized by the *P*. *beijingensis* GtfD and *A*. *chroococcum* GtfD enzymes. The *P*. *beijingensis* GtfD (A) and *A*. *chroococcum* GtfD (B) enzymes were incubated with malto-oligosaccharides (DP2-DP7), amylose V, and amylopectin at 37°C and pH 7.0 (*P*. *beijingensis* GtfD) or pH 6.5 (*A*. *chroococcum* GtfD) during 24 h. S, standard; G1, glucose; G2, maltose; G3, maltotriose; G4, maltotetraose; G5, maltopentaose; G6, maltohexaose; G7, maltoheptaose; AMV, amylose V; AMP, amylopectin; Pol, polymer.

The substrate specificity of the *P*. *beijingensis* GtfD was studied by incubating the enzyme with different carbohydrate substrates at 37°C for 24 h, and compared with that of the *A*. *chroococcum* GtfD enzyme (Fig 3). The *P*. *beijingensis* GtfD enzyme was inactive on sucrose, panose, nigerose, and isomalto-oligosaccharides with DP2, DP3, and DP5 (Data not shown), similar to the *A*. *chroococcum* GtfD and other 4,6-α-GTases [11,14,15]. Instead, the *P*.*beijingensis* GtfD enzyme catalyzed the conversion of malto-oligosaccharides (MOS) of DP3 to 7 showing both hydrolysis and transglycosylase (disproportionation) activity (Fig 3A). Indeed, incubation of *P*. *beijingensis* GtfD with MOS of DP3 to 7 revealed the formation of lower- and higher-molecular-mass products. Besides, with G4 and larger MOS as substrates, polymeric material was also clearly detected remaining at the origin of the TLC plates. However, the *P*. *beijingensis* GtfD failed to act on maltose. Similar substrate specificity was observed with *A*. *chroococcum* GtfD (Fig 3B). The main difference between both GtfD enzymes was observed when amylose V and amylopectin were used as substrates. As reported before, the *A*. *chroococcum* GtfD enzyme accumulated G2 and some low molecular mass oligosaccharides from these polymer substrates, reflecting its hydrolase/disproportionating activity. In contrast, these low molecular mass products were not clearly detectable by TLC when exploring the activity of *P*. *beijingensis* GtfD on amylose and amylopectin.

**Fig 4:**
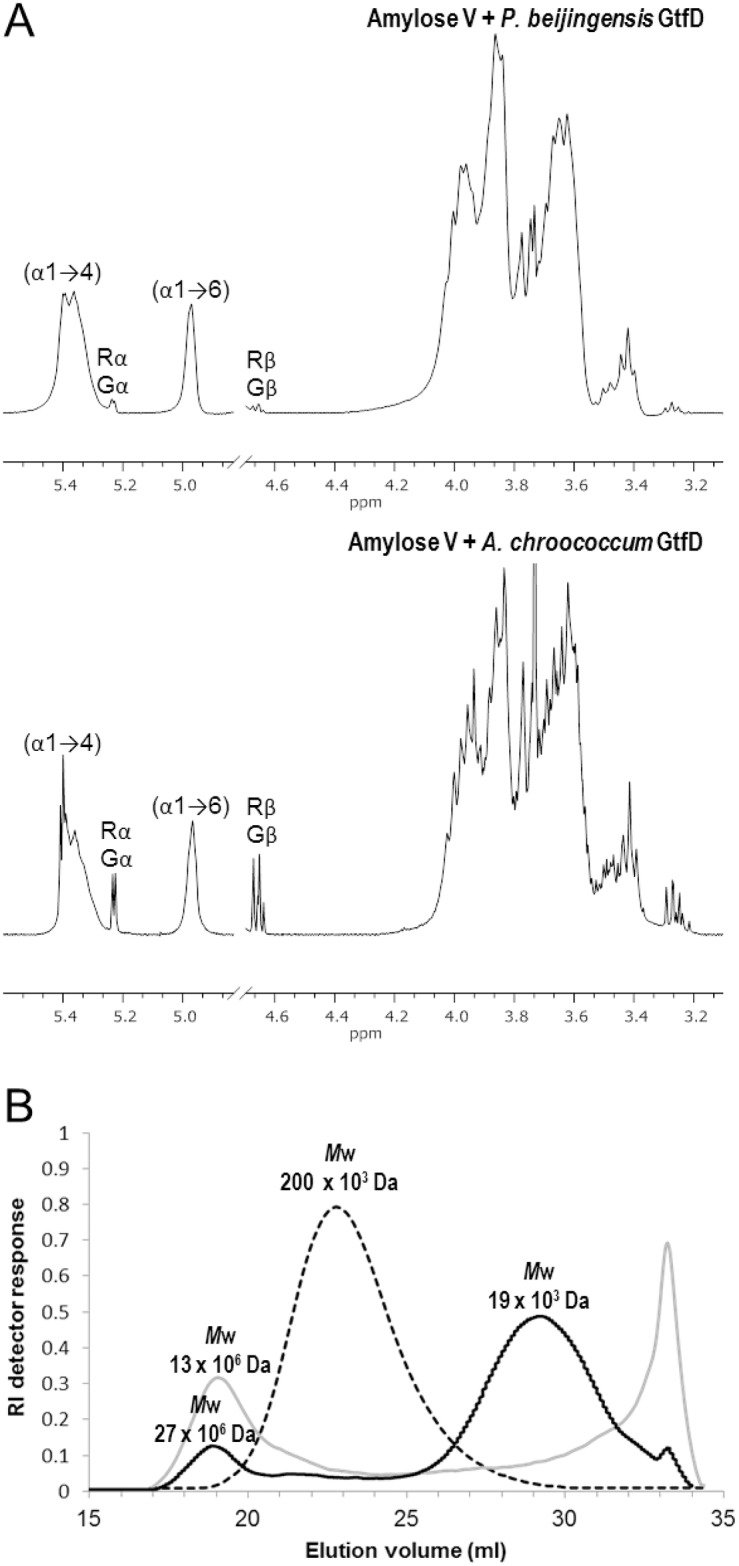
Structural analysis of the product mixtures generated after the incubation of amylose V with the *P*. *beijingensis* and *A*. *chroococcum* GtfD 4,6-α-GTase enzymes. (A) ^1^H NMR spectra of the product mixtures synthesized. The spectra were recorded in D_2_O at 298K. Chemical shifts are shown in parts per million relative to the signal of internal acetone (δ = 2.225). Gα/β and Rα/β indicate the anomeric signals corresponding to the D-Glc*p* units and the reducing -(1→4)-D-Glc*p* units, respectively. (B) HPSEC chromatograms of the product mixtures formed by the *P*. *beijingensis* GtfD enzyme and the *A*. *chroococcum* GtfD from amylose V. The dashed line corresponds to the elution profile of the starting amylose V. The solid black and grey lines correspond to the elution profiles of products synthesized by *P*. *beijingensis* and *A*. *chroococcum* GtfD enzymes, respectively.

^1^HNMR analysis of the product mixture generated from amylose V revealed the presence of two broad anomeric signals corresponding to the (α1→4) (δ ~ 5.40–5.35) and the newly formed (α1→6) linkages (δ ~ 4.97) (Fig 4A). This ^1^H NMR spectrum resembled that of the products derived from amylose V by *A*. *chroococcum* GtfD treatment suggesting that both GtfD enzymes have the same product specificity. The spectra also showed the presence of small signals corresponding to free glucose units (Gα H-1, δ 5.225; Gβ H-1, δ 4.637) and 4-substituted reducing end glucose residues (Rα H-1, δ 5.225; Rβ H-1, δ 4.652). These signals were much smaller in the case of the *P*. *beijingensis* GtfD product mixture reflecting that only trace amounts of glucose, maltose and other small oligosaccharides are present in this product, as previously observed by TLC analysis. The molar ratio of the (α1→4)-linked, (α1→6)-linked and reducing glucose residues for both reactions were nearly identical, and were 72:26:2 for the *A*. *chroococcum* GtfD and 75:25:<1, for the *P*. *beijingensis* GtfD. Methylation analysis of the product mixture synthesized by the *P*. *beijingensis* GtfD from amylose V revealed the presence of terminal, 4-substituted, 6-substituted, and 4,6-disubstituted glucopyranose residues in a molar percentage of 18, 56, 7 and 19%, in accordance with the linkage ratios determined by ^1^HNMR. This result confirmed that the *P*. *beijingensis* GtfD acts as a 4,6-α-glucanotransferase cleaving (α1→4) linkages and synthesizing a branched α-glucan consisting of (α1→4) and (α1→6) linkages.

Comparison of the products generated from amylose by the *P*. *beijingensis* and *A*. *chroococcum* GtfD enzymes by HPSEC with multi detection, revealed differences in their molecular mass distribution (Fig 4B). As reported before [15], after incubating amylose V with the *A*. *chroococcum* GtfD, the single peak corresponding to amylose with a small molecular mass (approximately 200 × 10^3^ Da) disappeared and two new peaks were formed: A peak eluting at ~ 19 ml corresponding to a high molecular mass (HMM) polymer with an average *M*_w_ of 13 x 10^6^, and a second peak eluting at ~ 34 ml corresponding to maltose and other small oligosaccharides. By contrast, the HPSEC profile of products synthesized by *P*. *beijingensis* GtfD from amylose V revealed the presence of two main polymer populations, whereas maltose and other small oligosaccharides were not significantly accumulated. Besides an early peak eluting at ~ 19 ml and corresponding to a HMM polymer with a *M*_w_ of 27 × 10^6^ Da, a second broad peak eluting at ~29 ml and corresponding to a low molecular mass (LMM) polymer with a *M*_w_ 19 × 10^3^ Da was detected. The elongation mechanism leading to the formation of this bimodal polymer molecular mass distribution remains to be investigated. The synthesis of HMM and LMM products may be the result of two distinct *processive* and *non-processive* elongation mechanisms in *P*. *beijingensis* GtfD, as described for the *B*. *subtilis* levansucrase [32]. Based on the refractive index response, the *P*. *beijingensis* GtfD HMM polymer represented only a small percentage (less than 20%) of the total product, the LMM polymer being the main product of the reaction.

### Characterization of the high- and low-molecular mass polymers produced by the *P*. *beijingensis* GtfD enzyme from amylose V

**Fig 5:**
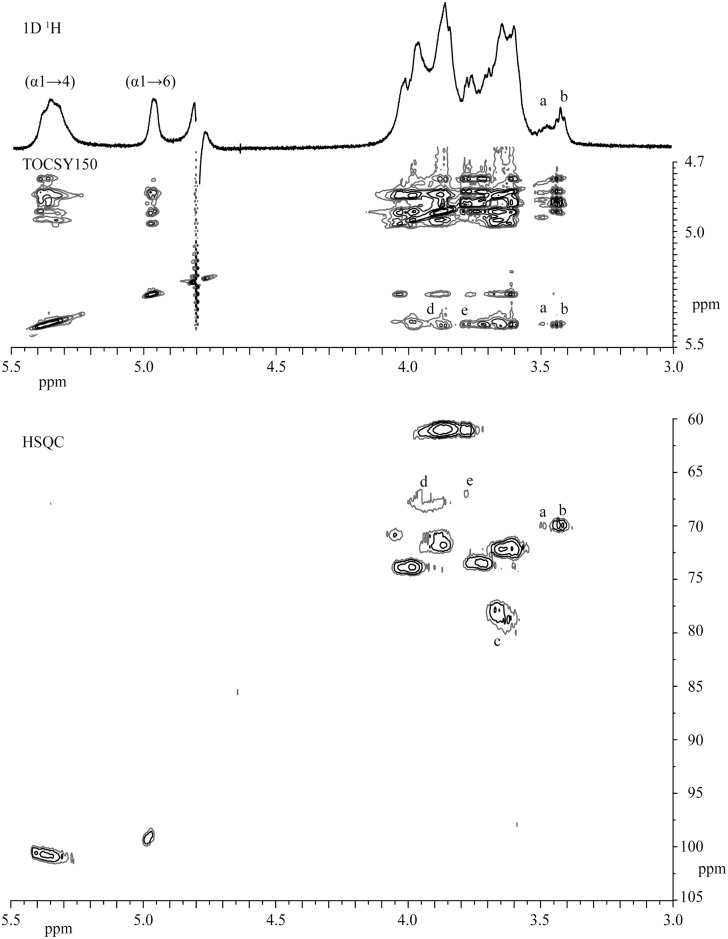
1D ^1^H NMR spectrum, 2D ^1^H-^1^H TOCSY spectrum (mixing time 150 ms), and 2D ^13^C-^1^H HSQC spectrum of the HMM polysaccharide produced by the *P*. *beijingensis* GtfD enzyme from amylose. The spectra were recorded in D_2_O at 298K. Peaks for (α1→4) and (α1→6) anomeric signals have been indicated. Structural reporter peaks a: H-4 for 6-substituted Glc*p*, b: H-4 for terminal Glc*p*, c: for H-4 for 4-substituted Glc*p*, d: H-6a for 6-substituted Glc*p* and e: H-6b for 6-substituted Glc*p*.

For a more detailed characterization the HMM and LMM polymers generated from amylose V by the *P*. *beijingensis* GtfD were isolated by size-exclusion chromatography analysis on Sephadex S-200 and subjected to 1D/2D ^1^H/^13^C NMR spectroscopy. As an example, Fig 5 presents 1D/2D ^1^H and ^13^C NMR spectra of the *P*. *beijingensis* GtfD HMM product. Very similar ^1^H NMR spectra were obtained for both polysaccharides, showing a linkage ratio (α1→4):(α1→6) = 71:29 for the HMM polymer and a linkage ratio (α1→4):(α1→6) = 77:23 for the LMM polymer, indicating a slight increase in the percentage of (α1→6)-linked glucose residues in the HMM polymer. 2D NMR data of the *P*. *beijingensis* GtfD α-glucans match those of the reuteran type of polymers generated by *A*. *chroococcum* GtfD and *L*. *reuteri* 121 GtfA glucansucrase from amylose and sucrose, respectively. Most notably, the typical chemical shift values corresponding to successive (α1→6) linkages were not identified in the 2D NMR spectra of *P*. *beijingensis* GtfD HMM (Fig 5) and LMM (not shown) products. The reuteran-like structure of the *P*. *beijingensis* GtfD products was further confirmed by methylation analysis, revealing the presence of terminal, 4-substituted, 6-substituted and 4,6-disubstituted glucopyranosyl units (Table 2). The HMM *P*. *beijingensis* GtfD product contains slightly less amounts of 6-substituted glucopyranosyl residues than the reuteran-like polymer synthesized by *A*. *chroococcum* GtfD. This results in a reuteran-like polymer with a slightly lower amount of alternating (α1→4)/(α1→6) glycosidic linkages (i.e. 11% rather than 18%), but a similar amount of branches (18%). Compared to the HMM *P*. *beijingensis* product, the LMM *P*. *beijingensis* GtfD product presents lower amounts of (α1→6) linkages in linear orientation reflected by the reduced amount of 6-substituted glucopyranosyl units (5% rather than 11%).

**Table 2:** Structural characterization of the HMM and LMM polymers synthesized by the *P*. *beijingensis* GtfD enzyme from amylose V. For comparison the characteristics of the polymer produced by the *A*. *chroococcum* GtfD enzyme are included as well.

Parameter	Type of glucosyl units	*A*. *chroococcum* GtfD polymer^c^	*P*. *beijingensis* GtfD HMM polymer	*P*. *beijingensis* GtfD LMM polymer
Methylation analysis (%)	Glc*p*(1→	19	17	15
	→4)-Glc*p*-(1→	45	54	62
	→6)-Glc*p*-(1→	18	11	5
	→4,6)-Glc*p*-(1→	18	18	18
NMR chemical shift (%)^a^	(α1→4)	68	71	77
	(α1→6)	32	29	23
Molecular mass (10^3^ Da)^b^		13 10^3^	27 10^3^	19

^a^ The data represent the ratios of integration of the surface areas of the (α1→6) linkage signal at 4.97 ppm and the (α1→4) linkage signal at 5.36 ppm in the ^1^H NMR spectra of the polysaccharides (see Fig 5).

^b^ The average molecular mass of polysaccharide was determined in duplicate.

^c^ Taken from Gangoiti *et al*., 2016 [15].

**Fig 6:**
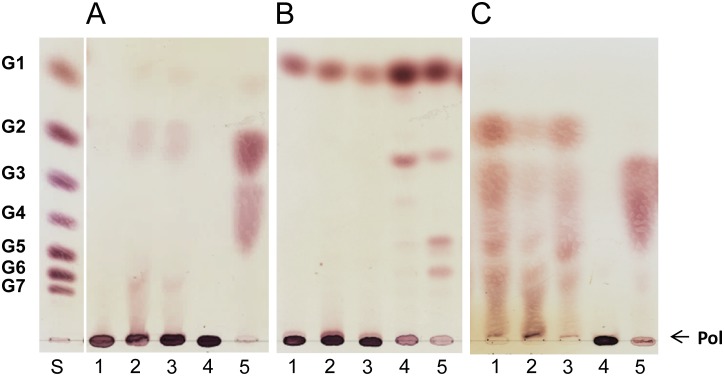
Enzymatic treatment of the *P*. *beijingensis* GtfD HMM and LMM polymers, *A*. *chroococcum* GtfD reuteran-like polymer, and *L*. *reuteri* 121 GtfB Isomalto/Malto-Polysaccharide (IMMP). Reaction mixtures containing 5 mg ml^-1^ of α-glucans were incubated separately with a high dose of (A) *Aspergillus oryzae* α-amylase, (B) *Chaetomium erraticum* dextranase and (C) *Klebsiella planticola* pullulanase M1 for 48 h at 37ᵒC and subjected to TLC analysis. Lanes 1–4: reaction products generated by the enzymatic treatment of the *P*. *beijingensis* GtfD HMM polymer, *P*. *beijingensis* GtfD LMM polymer, reuteran-like polymer, and IMMP, respectively. Lane 5, positive controls for the α-amylase, dextranase and pullulanase digestions: amylose (A), dextran (B) and pullulan (C). Lane S, standard: glucose (G1) to maltoheptaose (G7); Pol, polymer.

**Fig 7:**
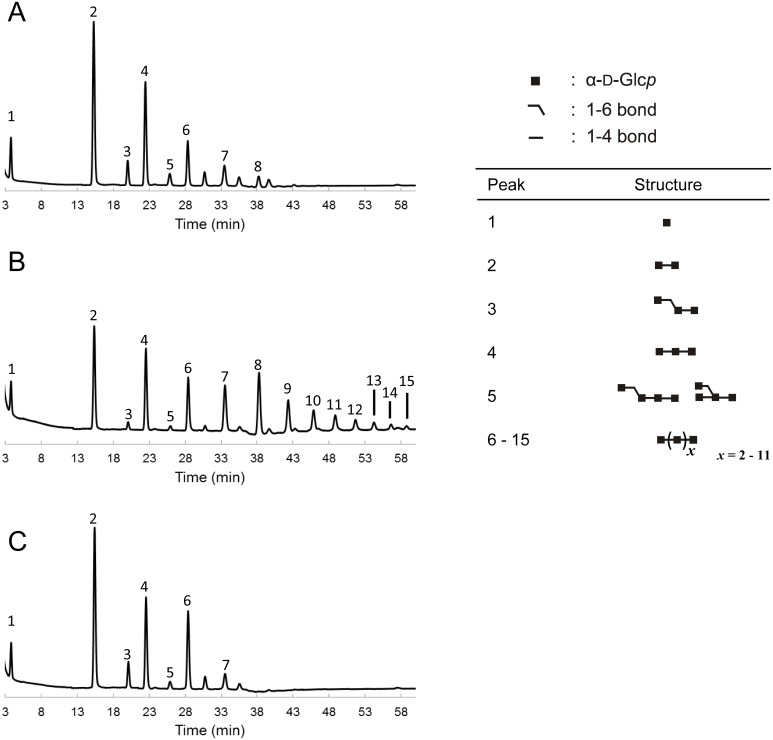
HPAEC profiles of the oligosaccharides formed after treatment of the *P*. *beijingensis* GtfD and *A*. *chroococcum* GtfD polymers with pullulanase M1. The *P*. *beijingensis* GtfD HMM polymer (A), *P*. *beijingensis* GtfD LMM polymer (B), and *A*. *chroococcum* GtfD (C) were incubated with an excess of pullulanase M1 for 48 h at 37°C and pH 5. Established oligosaccharide structures are included. The identity of peaks 1–16 was assigned using commercial oligosaccharide standards and by comparison with the profile of the pullulanase hydrolysate of reuteran [17].

To gain more insight into the carbohydrate structures of the HMM and LMM *P*. *beijingensis* GtfD products, and to compare them with the *A*. *chroococcum* GtfD reuteran-like polymer and *L*. *reuteri* 121 GtfB IMMP, these α-glucans were incubated for 48 h with different hydrolytic enzymes: α-amylase, dextranase and pullulanase M1 (Fig 6). Examination of the hydrolysis products showed that HMM *P*. *beijingensis* GtfD and *A*. *chroococcum* GtfD polymers were resistant to the endo-α-1,4-hydrolase activity of the α-amylase. In both cases, only trace amounts of HMM oligosaccharides and maltose were detected after 48 h of α-amylase digestion. The *P*. *beijingensis* LMM product, however, appeared to be slightly more susceptible to α-amylase digestion, as revealed by the decreased intensity of the spot corresponding to the polymeric material and the accumulation of HMM oligosaccharides. This result correlates well with the decreased molecular mass and lower amount of (α1→6) linkages of the LMM *P*. *beijingensis* GtfD product, compared to its HMM counterpart. As reported before, the IMMP GtfB product was also resistant to the action of the α-amylase, whereas the amylose substrate was completely degraded under the same conditions. Both *P*. *beijingensis* GtfD products and the *A*. *chroococcum* GtfD were resistant to the endo-α-1,6-hydrolase activity of dextranase, reflecting the absence of consecutive (α1→6) linkages in these polymers. In contrast, IMMP and dextran, which contain a linear backbone of (α1→6)-linked D-glucopyranosyl repeating units were efficiently hydrolyzed by the action of dextranase. Pullulanase specifically hydrolyses the (α1→6) linkages of pullulan, amylopectin, and other 4,6-branched polysaccharides. After treatment with pullulanase, the *P*. *beijingensis* GtfD and the *A*. *chroococcum* GtfD products were degraded into smaller oligosaccharides, reflecting the presence of alternating (α1→6)/(α1→4), and (α1→4,6) branching points in these polymers. IMMP was not hydrolyzed by the action of the pullulanase, which is in agreement with the presence of linear (α1→6) chains in its structure. More details of the precise structures of the *P*. *beijingensis* GtfD oligosaccharide products formed upon incubation of the HMM and LMM polymers with pullulanase M1 were obtained by their analysis by HPAEC (Fig 7). Incubation of the HMM *P*. *beijingensis* GtfD polymer with pullulanase yielded a mixture of MOS up to DP6 (Fig 7A), whereas in the case of the LMM *P*. *beijingensis* GtfD polymer additional peaks corresponding to MOS up to DP13 were also identified (Fig 7B). As reported before the digestion of the *A*. *chroococcum* GtfD polymer resulted in the formation of MOS of DP2 to 5 (Fig 7C) and confirmed that this α-glucan consists of maltose, maltotriose, maltotetraose and maltopentaose units connected via single (α1→6) bonds in linear or branched orientations. The identification of MOSs with higher DPs in the case of the HMM and LMM *P*. *beijingensis* GtfD products leads to structures containing longer linear (α1→4) sequences.

### Composite models

**Fig 8:**
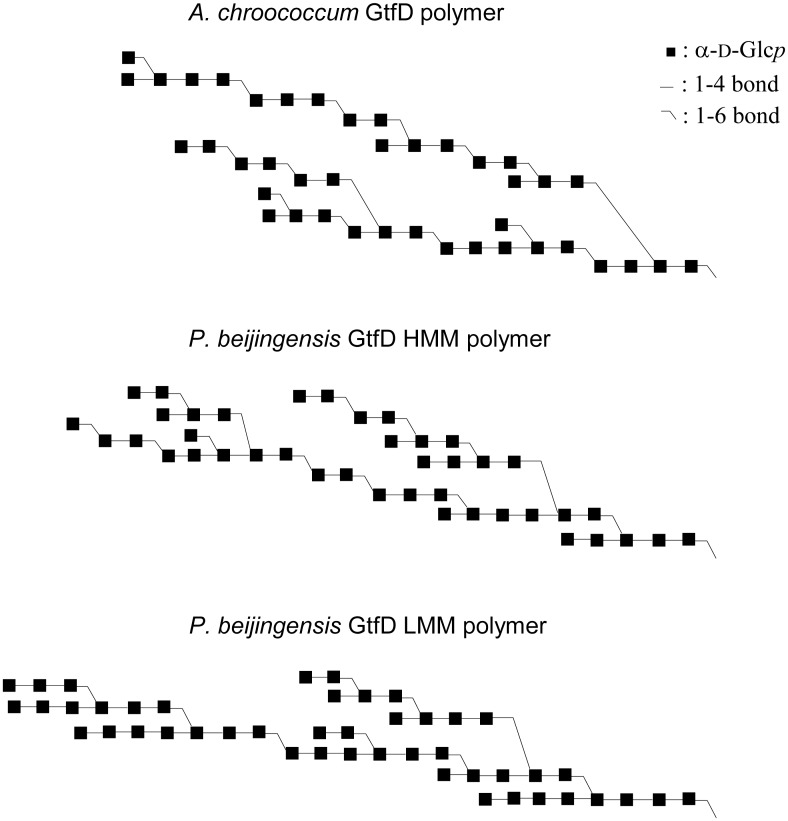
Visual representation of composite structures for HMM and LMM *P*. *beijingensis* GtfD polymers formed from amylose V. The composite structures contain all structural features established for the respective products. Quantities of each structural element fit with the combined data of 1D ^1^H NMR integration and methylation analysis, as well as enzymatic degradation studies with α-amylase, dextranase and pullulanase. For comparison the composite structure for the *A*. *chroococcum* GtfD polymer from amylose is represented as well. [15]

Using the data obtained by methylation analysis, NMR spectroscopy and enzymatic digestion studies composite models were constructed, reflecting all major structural elements observed for the HMM and LMM *P*. *beijingensis* GtfD products (Fig 8). Compared with the *A*. *chroococcum* GtfD product [15] the linear (α1→4)-linked sequences are longer in the *P*. *beijingensis* GtfD HMM polymer (up to DP6 in the model) and even longer in the *P*. *beijingensis* GtfD LMM polymer (up to DP8 in the model). Although longer linear DPs of consecutive (α1→4) (DP13) are observed after treating the LMM *P*. *beijingensis* GtfD product with pullulanase, the amounts are too low to be reflected in the composite model. For the *A*. *chroococcum* GtfD product the pullulanase digestion showed only up to DP5 linear (α1→4)-linked sequences.

### Oligosaccharides formed in time by the *P*. *beijingensis* GtfD enzyme from maltoheptaose

**Fig 9:**
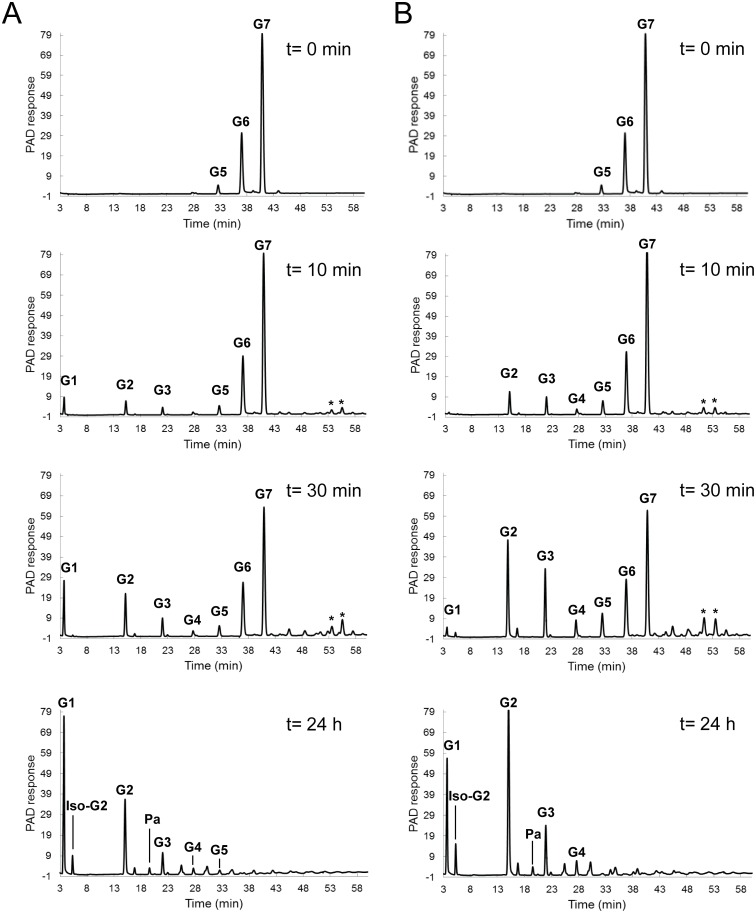
HPAEC profiles of the oligosaccharides formed in time by the *P*. *beijingensis* GtfD and *A*. *chroococcum* GtfD enzymes from maltoheptaose. Reaction mixtures containing 25 mM maltoheptaose were incubated with 20 μg ml^-1^ of *P*. *beijingensis* GtfD (A) and *A*. *chroococcum* GtfD (B) enzymes for t = 10 min, 30 min, 3 h, and 24 h, at 37°C and pH 7.0 and pH 6.5, respectively. The identity of peaks was assigned using commercial oligosaccharide standards. * Unidentified carbohydrate structures. G1, glucose; G2-G6, maltose to maltohexaose; iso-G2, isomaltose; Pa, panose.

To gain a better understanding of the reaction mechanism of the *P*. *beijingensis* and *A*. *chroococcum* GtfD enzymes, both enzymes were incubated with maltoheptaose (G7), and the oligosaccharides formed in time were analyzed by HPAEC (Fig 9). Incubation of G7 (slightly contaminated with G6 and G5) with the *P*. *beijingensis* GtfD enzyme yielded G1, G2 and two peaks corresponding to compounds of unknown structure with a higher DP eluting at 53.5 and 55.5 min at the early stage of the reaction (Fig 9A). A small peak corresponding to G3 was also identified, whereas the amounts of G5 and G6 remained low. HPAEC analysis of MOS standards of DPs from 2 to 30 revealed that these two peaks of unidentified structure eluted slightly earlier than maltododecaose (G12) and maltotridecaose (G13), suggesting structures with DP of 12 and 13 and at least one (α1→6) linkage. The deficit observed in the G5 and G6 released, together with the formation of G1 and G2 indicates that the *P*. *beijingensis* GtfD enzyme catalyzes the transfer of maltopentaosyl- and maltohexaosyl- moieties to a G7 acceptor substrate, yielding the two unknown peaks (peaks eluting at 53.5 and 55.5 min). After 24 h, the unknown oligosaccharides initially formed by the *P*. *beijingensis* GtfD enzyme disappeared suggesting that these compounds can be subsequently used as donor and/or acceptor substrates. When exploring the activity of *A*. *chroococcum* GtfD, G2, G3 and two unknown compounds with high DP eluting at 51.4 and 53.5 min, were detected as the first clear products formed from G7 (Fig 9B). The appearance of G2 and the peak eluting at 53.5 min, which was also observed in the case of *P*. *beijingensis* GtfD, indicates that the *A*. *chroococcum* GtfD also has the ability to catalyze a maltopentaosyl-transfer reaction from G7. The excess of G3, compared to G4, together with the identification of a peak eluting at 51.4 min, suggests that *A*. *chroococcum* GtfD is also able to cleave off a maltotetraosyl unit and transfer it to a MOS acceptor molecule. Most notably, the release of G1 as a side product of the maltohexaosyl-unit transfer reaction was not seen for the *A*. *chroococcum* GtfD during the early stage of the reaction. In agreement with this mode of action, the *A*. *chroococcum* GtfD activity on amylose results in the synthesis of a reuteran-like polymer built-up from MOS up to DP5 linked by (α1→6) linkages. The preference for the transfer of longer glucan chains by the *P*. *beijingensis* GtfD enzyme is also reflected by the presence of longer linear (α1→4) sequences in the structure of its reuteran-like products. Overall these results indicate that the architecture of the active site of these GtfD type of enzymes may present more than one donor binding subsite, similar to other starch-converting enzymes of the evolutionary related GH13 and GH77 families [5,33,34]. As a result, these GtfD enzymes have the ability to transfer MOS units, differing from GSs that strictly transfer a single glucose unit per reaction cycle. Differences in the number of donor substrate binding subsites may explain the differences observed in the length of the chains transferred by the *P*. *beijingensis* and the *A*. *chroococcum* GtfD enzymes.

### *In vitro* digestibility of polymers produced by the *P*. *beijingensis* GtfD enzyme from amylose V and wheat starch

**Fig 10:**
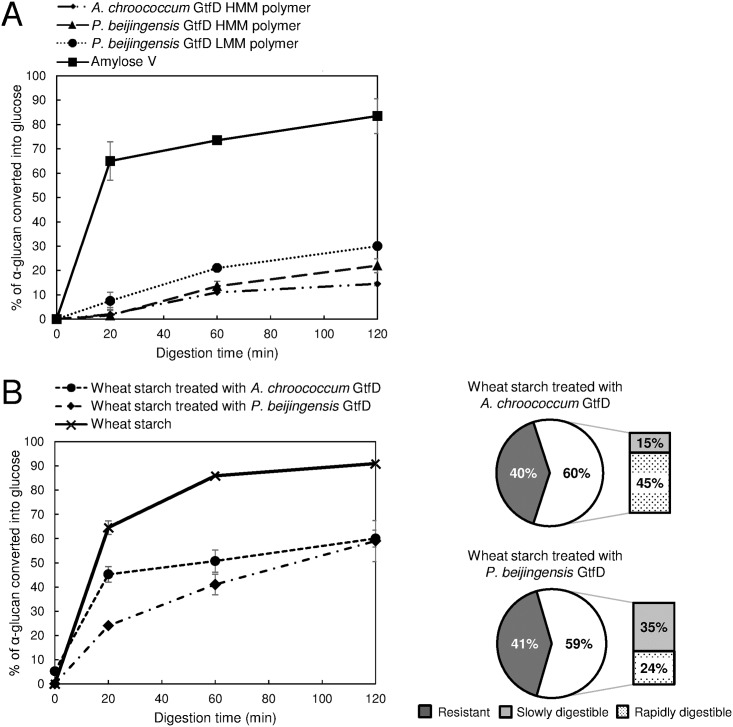
*In vitro* digestibility of the *P*. *beijingensis* GtfD and *A*. *chroococcum* GtfD α-glucan products in time. Reaction mixtures containing 1 mg ml^-1^ of α-glucan samples were incubated with 100 U ml^-1^ of porcine pancreatin and rat intestinal powder extracts, concurrently, at 37°C. (A) Digestibility of the HMM and LMM polymers synthesized by the *P*. *beijingensis* GtfD and *A*. *chroococcum* GtfD enzymes from amylose V compared to the amylose V starting substrate (B) Digestibility of the gelatinized wheat starch before and after treatment with *P*. *beijingensis* GtfD and *A*. *chroococcum* GtfD enzymes. The product mixtures were obtained from 0.6% w v^-1^ gelatinized wheat starch by incubations with 4.6 μg ml^-1^ of the GtfD 4,6-α-GTase enzymes for 24 h at 37°C. The amounts of resistant (undigested after 120 min), slowly digestible (digested between 20 and 120 min) and rapidly digestible (digested within the first 20 min) carbohydrates present in the *P*. *beijingensis* GtfD- and *A*. *chroococcum* GtfD-treated wheat starches are indicated.

The polymers generated from amylose V by the *P*. *beijingensis* GtfD and *A*. *chroococcum* GtfD were subjected to hydrolysis by a combination of porcine pancreatin and rat intestinal powder extracts to simulate human small intestinal digestion. In *in vitro* simulations of starch digestibility, porcine pancreatic α-amylase is commonly used as a surrogate for human pancreatic α-amylase [35] and, more recently, rat intestinal powder extracts have replaced the fungal amyloglucosidase because their α-glucosidic activity more closely resembles that of human intestinal enzymes [36]. As shown in Fig 10A, the rate and extent of hydrolysis of the *A*. *chroococcum* GtfD polymer and the *P*. *beijingensis* GtfD HMM and LMM polymers were significantly lower compared to that of the amylose V starting substrate. Only 15, 22, and 30% of the HMM *A*. *chroococcum* GtfD polymer, HMM *P*. *beijingensis* GtfD polymer and LMM *P*. *beijingensis* GtfD polymer, respectively, were hydrolyzed to glucose after 120 min of reaction. Although the HMM polymers synthesized by the *P*. *beijingensis* and *A*. *chroococcum* GtfD enzymes were digested at similar rates during the first 60 min of the simulated digestion, differences were observed in the second half of the reaction. At later stages (from 60 to 120 min), the HMM *A*. *chroococcum* GtfD polysaccharide was not significantly hydrolyzed, whereas in the case of the HMM *P*. *beijingensis* GtfD polymer, the amount of hydrolyzed glucan increased from 13% to 22% indicating that this polysaccharide is slightly more susceptible to hydrolysis by digestive enzymes than the HMM *A*. *chroococcum* GtfD product. Previous studies have demonstrated that the fine structure of the α-glucans is a determining factor of their digestibility. Specifically, an enhanced (α1→6) branch density was found to lead to a slower *in vitro* digestion rate [37,38]. Also, an increased amount of consecutive (α1→6) linkages resulted in a higher resistance to digestion by rat intestinal enzymes [13]. Our data suggest for the first time that the digestibility of the α-glucans is also inversely proportional to the amount of alternating (α1→6)/(1→4) linkages present in the polymers.

The impact of the direct action of the GtfD enzymes on starch digestibility was also evaluated. Interestingly, the enzymatic modification of the gelatinized wheat starch with the *P*. *beijingensis* and *A*. *chroococcum* GtfD enzymes resulted in a slower rate and reduced level of hydrolysis (Fig 10B). For both GtfD-treated starches, only 60% of the products were hydrolyzed to glucose after 120 min of simulated intestinal digestion, indicating that both enzymes produce similar amounts of α-glucans resistant to the digestibility treatment. However, the hydrolysis profiles of the *A*. *chroococcum* GtfD- and *P*. *beijingensis* GtfD-treated starches showed significant differences. Whereas most of the digestible fraction of the *A*. *chroococcum* GtfD-treated starch was hydrolyzed to glucose after 20 min, the rate of hydrolysis for *P*. *beijingensis* GtfD-treated starch continually increased over the 120 min of simulated intestinal digestion. These results indicate that the *A*. *chroococcum* GtfD- and *P*. *beijingesis* GtfD-treated starches have different amounts of rapidly- and slowly-digestible carbohydrates, defined as the α-glucans that are digested within the first 20 min and between 20 and 120 min of the reaction, respectively (Fig 10B). The higher content of rapidly-digestible carbohydrates in the case of the *A*. *chroococcum* GtfD-treated starch (45% rather than 24%) is in agreement with the higher release of maltose and other LMM malto-oligosaccharides observed as a result of the activity of this enzyme. Considering the fast digestion rate of the untreated-gelatinized wheat starch, and also its low content in carbohydrates resistant to the *in vitro* digestion procedure (~10%), both GtfD enzymes may be suitable biocatalysts for the conversion of the starch present in food into carbohydrates with reduced glucose released. However, based on the observed slow digestibility property of the *P*. *beijingensis* GtfD-treated starch, this enzyme seems to be a preferred biocatalyst to reduce the glycemic index of starch-containing food.

## Supporting information

S1 Fig GenBank accession numbers of the family GH13 and GH70 protein sequences used in the phylogenetic tree of Fig 1. Note that the GtfD-like protein encoded by *Burkholderia* sp. NFACC38-1 is annotated by its IMG/ER Gene ID.
(TIF)

S1 Table GtfD-, GtfC- and GtfB-like sequences identified via a BLASTp search using the *Azotobacter chroococcum* NCIMB 8003 GtfD enzyme as query. GtfD-like and GtfC-like sequences are shown in bold and lightly shaded, respectively. GtfB-like sequences are displayed in grey.
(DOCX)
