# Supplementary figures and images for: Characterization of the Paenibacillus beijingensis DSM 24997 GtfD and its glucan polymer products representing a new glycoside hydrolase 70 subfamily of 4,6-α-glucanotransferase enzymes
Source: PLoS One. 2017 Apr 11;12(4):e0172622. doi: 10.1371/journal.pone.0172622 (PMC5388325; doi:10.1371/journal.pone.0172622)

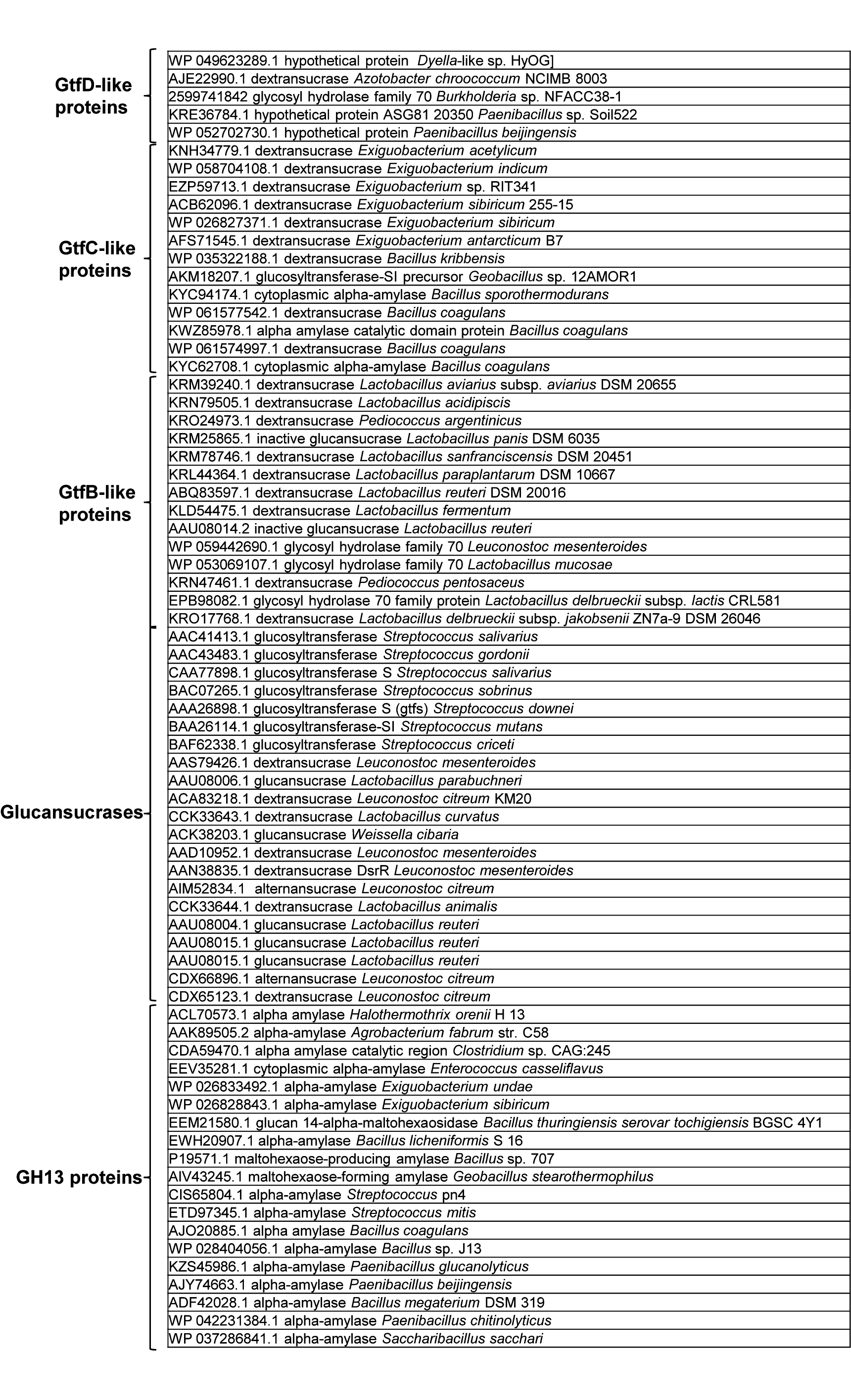

Supplement: S1 Fig — Note that the GtfD-like protein encoded by Burkholderia sp. NFACC38-1 is annotated by its IMG/ER Gene ID. (TIF) [file pone.0172622.s001.tif]
