# Supplementary material for: Characterization of the Paenibacillus beijingensis DSM 24997 GtfD and its glucan polymer products representing a new glycoside hydrolase 70 subfamily of 4,6-α-glucanotransferase enzymes
Source: PLoS One. 2017 Apr 11;12(4):e0172622. doi: 10.1371/journal.pone.0172622 (PMC5388325; doi:10.1371/journal.pone.0172622)
Supplement: S1 Table — GtfD-like and GtfC-like sequences are shown in bold and lightly shaded, respectively. GtfB-like sequences are displayed in grey. (DOCX) [file pone.0172622.s002.docx]

| **Organism** | **Length** | **Coverage** | **Identity** | **Origin** | **Accession^a^** |
| --- | --- | --- | --- | --- | --- |
| ***Azotobacter chroococcum* NCIMB 8003** | **780** | **100%** | **100%** | **Soil** | **AJE22990.1** |
| ***Dyella*-like sp. HyOG** | **790** | **98%** | **71%** | **Insect *Hyalesthes obsoletus* gut** | **WP_049623289.1** |
| ***Paenibacillus beijingensis* DSM 24997** | **776** | **86%** | **48%** | **Rhizospere soil of jujube** | **WP_052702730.1** |
| ***Burkholderia* sp. NFACC38-1** | **686** | **97%** | **46%** | **Root endophyte of switchgrass** | **2599741842** |
| ***Paenibacillus* sp. Soil522** | **576** | **67%** | **45%** | ***Arabidopsis thaliana* isolate** | **WP_056638435.1** |
| *Bacillus coagulans* B4098 | 751 | 87% | 42% | Chinese tomato | WP_061566291.1 |
| *Bacillus coagulans* XZL4 | 742 | 87% | 42% | Soil near a corncob factory | WP_029141257.1 |
| *Bacillus coagulans* B4098 | 751 | 87% | 42% | Chinese tomato | WP_061577542.1 |
| *Bacillus coagulans* B4096 | 889 | 87% | 42% | Tomato supreme | KYC80659.1 |
| *Bacillus coagulans* B4099 | 759 | 87% | 42% | Indian curry | WP_061574997.1 |
| *Bacillus coagulans* H-1 | 876 | 87% | 42% | Soil | WP_017553304.1 |
| *Bacillus coagulans* 2-6 | 954 | 87% | 42% | Soil | AEH52441.1 |
| *Bacillus coagulans* GED7749B | 889 | 87% | 42% | Human urogenital tract | KWZ85978.1 |
| *Bacillus coagulans* DSM 1 = ATCC 7050 | 965 | 87% | 42% | Evaporated milk | AJH79253.1 |
| *Bacillus coagulans* B4100 | 954 | 87% | 42% | Low pH sauce | KYC62708.1 |
| *Bacillus sporothermodurans* B4102 | 902 | 87% | 41% | Indian curry | KYC94174.1 |
| *Geobacillus* sp. 12AMOR1 | 903 | 87% | 42% | Deep-sea hydrothermal vent site | AKM18207.1 |
| *Bacillus kribbensis* DSM 17871 | 904 | 87% | 40% | Soil | WP_035322188.1 |
| *Exiguobacterium sibiricum* 7-3 | 893 | 87% | 39% | Permafrost soil | WP_026827371.1 |
| *Exiguobacterium antarcticum* B7 | 893 | 87% | 40% | Biofilm in Ginger lake (Antarctica) | WP_014971370.1 |
| *Exiguobacterium antarcticum* DSM 14480 | 893 | 87% | 40% | Microbial mat (Antarctica) | WP_026830256.1 |
| *Exiguobacterium indicum* RSA42 | 892 | 87% | 39% | Hamta glacier (India) | WP_058704108.1 |
| *Exiguobacterium acetylicum* DSM 20416 | 892 | 87% | 39% | Creamery waste | WP_029342707.1 |
| *Exiguobacterium* sp. ZWU0009 | 892 | 87% | 40% | Zebrafish intestine | WP_047390368.1 |
| *Exiguobacterium sibiricum* 255-15 | 893 | 87% | 39% | Permafrost sediment | WP_012371512.1 |
| *Exiguobacterium undae* DSM 14481 | 893 | 87% | 39% | Garden pond | WP_028105602.1 |
| *Exiguobacterium acetylicum* ZBG2 | 892 | 87% | 39% | Soil isolate | WP_050678213.1 |
| *Exiguobacterium* sp. KKBO11 | 892 | 87% | 40% | Surface Water | OAI88704.1 |
| *Exiguobacterium* sp. RIT341 | 892 | 87% | 40% | Endophyte isolated from Salix | WP_035410561.1 |
| *Pediococcus pentosaceus* DSM 20336 | 883 | 63% | 36% | Dried American beer yeast | KRN47461.1 |
| *Lactobacillus mucosae* LM1 | 881 | 63% | 36% | Piglet feces | WP_053069107.1 |
| *Pediococcus pentosaceus* IE-3 | 926 | 63% | 36% | Dairy industry effluent | WP_002833996.1 |
| *Lactobacillus plantarum* subsp. *argentoratensis* DSM 16365 | 880 | 0.63 | 0.36 | Fermented cassava roots (fufu) | KRL97820.1 |
| *Lactobacillus plantarum* WLPL04 =Nizo2753 | 922 | 63% | 36% | Sourdough fermented | WP_057138784.1 |
| *Lactobacillus delbrueckii* subsp. *Sunkii* JCM 17838 | 922 | 63% | 36% | Isolated from sunki | WP_050952694.1 |
| *Lactobacillus plantarum* Nizo2889 | 974 | 63% | 36% | Banana fermented | KZU77932.1 |
| *Lactobacillus plantarum* AG30 | 932 | 63% | 36% | Sheep rumen | WP_033607967.1 |
| *Lactobacillus plantarum* Nizo2753 | 1142 | 63% | 36% | Sourdough fermented | KZU41702.1 |
| *Lactobacillus delbrueckii* subsp. *jakobsenii* ZN7a-9 = DSM 26046 | 895 | 63% | 36% | Malted sorghum wort | KRO17768.1 |
| *Lactobacillus delbrueckii* subsp. *lactis* DSM 20072 | 1252 | 63% | 36% | Emmental cheese | WP_035182758.1 |
| *Lactobacillus delbrueckii* subsp. *jakobsenii* ZN7a-9 = DSM 26046 | 948 | 63% | 36% | Malted sorghum wort | WP_057727099.1 |
| *Lactobacillus delbrueckii* subsp. *Lactis* | 922 | 63% | 36% | Information not available | WP_057709472.1 |
| *Lactobacillus delbrueckii* subsp. *Lactis* | 852 | 63% | 36% | Information not available | WP_035162295.1 |
| *Lactobacillus salivarius* GJ-24 | 852 | 64% | 35% | Healthy adult intestine | WP_050809355.1 |
| *Lactobacillus delbrueckii* DSM 15353 | 1143 | 63% | 36% | Fermented fish | WP_002879779.1 |
| *Lactobacillus delbrueckii* subsp. *lactis* CRL 58 | 922 | 63% | 36% | Hard cheese | EPB98082.1 |
| *Lactobacillus delbrueckii* subsp. *bulgaricus* ND02 | 1294 | 63% | 36% | Human intestinal tract | WP_013439942.1 |
| *Lactobacillus delbrueckii* subsp. *bulgaricus* PB2003/044-T3-4 | 957 | 63% | 36% | Human urogenital tract | EFK31460.1 |
| *Leuconostoc mesenteroides* 406 | 850 | 83% | 30% | Fermented mare milk | WP_059442690.1 |
| *Lactobacillus paraplantarum* DSM 10667 | 920 | 84% | 29% | Fermented beverages | KRL44364.1 |
| *Lactobacillus salivarius* GJ-24 | 1626 | 64% | 35% | Healthy adult intestine | EGM52218.1 |
| *Lactobacillus acidipiscis* KCTC 13900 | 1160 | 71% | 36% | Halloumi cheese | WP_056988183.1 |
| *Lactobacillus acidipiscis* DSM 15353 | 1143 | 71% | 36% | Cheese, Halloumi | KRN79505.1 |
| *Lactobacillus delbrueckii* subsp. *delbrueckii* DSM 20074 | 1294 | 63% | 36% | Sour grain mash | WP_057717954.1 |
| *Lactobacillus plantarum* CIP104448 | 1266 | 74% | 36% | Human stool | WP_052661628.1 |
| *Lactobacillus delbrueckii* subsp. *delbrueckii* KACC 13439 | 965 | 63% | 35% | Information not available | WP_052933722.1 |
| *Lactobacillus plantarum* Nizo3893 | 1421 | 74% | 36% | Human stool | KZU83671.1 |
| *Lactobacillus delbrueckii* subsp. *delbrueckii* DSM 20074 | 997 | 63% | 36% | Sour grain mash | WP_025895575.1 |
| *Lactobacillus plantarum* Nizo3893 | 1480 | 74% | 36% | Human stool | WP_063488851.1 |
| *Lactobacillus plantarum* NL42 | 907 | 84% | 28% | Chinese dairy product | WP_052697219.1 |
| *Lactobacillus plantarum* 16 | 1348 | 84% | 29% | Malt production steep water | WP_016526729.1 |
| *Pediococcus argentinicus* DSM 23026 | 890 | 65% | 34% | Fermented wheat flour | KRO24973.1 |
| *Lactobacillus sanfranciscensis* DSM 20451 | 1151 | 87% | 28% | Sourdough | KRM78746.1 |
| *Pediococcus argentinicus* DSM 23026 | 898 | 65% | 34% | Fermented wheat flour | WP_057799472.1 |
| *Lactobacillus plantarum* Nizo1840 | 895 | 65% | 34% | Cereal fermented (Ogi) | KZT79262.1 |
| *Lactobacillus reuteri* TMW1.656 | 1620 | 63% | 35% | Sourdough | KOF04763.1 |
| *Lactobacillus reuteri* TMW1.106 | 1383 | 63% | 35% | Sourdough | ABP88725.1 |
| *Lactobacillus reuteri* ML-1 | 1620 | 65% | 35% | Information not available | AAU08003.2 |
| *Lactobacillus reuteri* 121 | 1619 | 63% | 34% | Pig gut | AAU08014.2 |
| *Lactobacillus reuteri* JCM 1112 | 1488 | 63% | 34% | Human feces | WP_003668618.1 |
| *Lactobacillus reuteri* DSM 20016 | 1363 | 63% | 34% | Intestine of adult | ABQ83597.1 |
| *Lactobacillus panis* DSM 6035 | 1603 | 65% | 33% | Sourdough | KRM25865.1 |
| *Lactobacillus reuteri* mlc3 | 1488 | 63% | 34% | Rodent host | WP_019251413.1 |
| *Lactobacillus aviarius* subsp. *aviarius* DSM 20655 | 1691 | 81% | 29% | Faeces of chicken | KRM39240.1 |
| *Lactobacillus reuteri* pg-3b | 1622 | 63% | 34% | Pig host | CUR36485.1 |
| *Lactobacillus fermentum* ATCC 14931 | 1014 | 66% | 34% | Human gastrointestinal-tract | WP_003683900.1 |
| *Lactobacillus fermentum* 28-3-CHN | 986 | 66% | 34% | Human urogenital /tract | WP_004563243.1 |
| *Lactobacillus fermentum* strain 39 | 1478 | 66% | 34% | Oral cavity of healthy individuals | WP_046948074.1 |
| *Pediococcus damnosus* TMW 2.1533 | 1409 | 65% | 33% | Brewery environment | WP_062904623.1 |
| *Pediococcus damnosus* TMW 2.1535 | 1469 | 65% | 33% | Pilsner beer | WP_062916828.1 |
| *Pediococcus damnosus* TMW 2.1533 | 1134 | 65% | 33% | Brewery environment | AMV63808.1 |
| *Pediococcus damnosus* TMW 2.1535 | 1169 | 65% | 33% | Pilsner beer | AMV68182.1 |

^a^ The protein sequences are annotated by their GenBank Accession number, except for the *Burkholderia* sp. NFACC38-1 GtfD-like protein sequence that is labeled with its IMG/ER Gene ID.
